# Evaluation of Characteristics and Outcomes for Patients with Diabetic Ketoacidosis (DKA) With and Without COVID-19 in Elmhurst Queens During Similar Three-Month Periods in 2019 and 2020

**DOI:** 10.7759/cureus.16427

**Published:** 2021-07-16

**Authors:** Urja Patel, Linda Deluxe, Carlos Salama, Aaron Ross Jimenez, Adrian Whiting, Cedrick Lubin, Nancy Tarlin

**Affiliations:** 1 Internal Medicine, Mount Sinai Elmhurst Hospital, New York, USA; 2 Endocrinology, Mount Sinai Elmhurst Hospital, New York, USA; 3 Internal Medicine, Icahn School of Medicine at Mount Sinai, New York, USA; 4 Endocrinology, Icahn School of Medicine Mount Sinai / NYC Health + Hospitals/Elmhurst, New York City, USA

**Keywords:** diabetic ketoacidosis, glycated hemoglobin (hba1c), mortality, diabetes, covid-19

## Abstract

Aim

There are reports of COVID-19 patients presenting with new onset diabetes, diabetic ketoacidosis (DKA), and hyperosmolar hyperglycemic syndrome (HHS). We compared the characteristics of patients with DKA with and without COVID-19 and their effect on mortality.

Research design and methods

A retrospective study at Elmhurst Hospital Center in Queens, New York was performed using ICD-10 codes to identify patients with DKA from March 1 to May 31 in 2019 and 2020.

Results

While comparing COVID-19 patients with DKA to the DKA patients without COVID-19 in both 2019 and 2020, hispanic patients, males, and type 2 diabetes predominated. COVID-19 patients were older (p=0.010), had more hypertension (p=0.002), and severe lactic acidosis (p=0.006). Mortality for DKA patients with COVID-19 was 57%, for DKA patients without COVID-19 it was 2.1% (p=0.0001), and for diabetic patients (no DKA) with COVID-19 it was 39% (p=0.035). Within the COVID-19 group, older age (mean age 65), (p=0.014), elevated CRP (p=0.012), low O_2_ saturation (p=0.001), and beta blocker use (P=0.01) were associated with increased mortality.

Conclusions

COVID-19 patients are older, have a history of hypertension, more severe DKA and lactic acidosis than patients without COVID-19. There was no increase in DKA with HHS. DKA, but not diabetic parameters, hypertension, and older age predicted a poor outcome.

## Introduction

Coronavirus disease (COVID-19), caused by severe acute respiratory syndrome corona virus 2 (SARS-CoV-2), is a global pandemic that has caused several million deaths worldwide [1,2]. The first COVID-19 cases were reported in Wuhan, China in December 2019 [2,3]. The virus is highly contagious, causing mild to severe viral illness and, at the extreme spectrum, acute respiratory distress syndrome (ARDS) and multi-organ system failure [3,4,5]. Men, older patients, and patients with certain comorbidities, such as hypertension [6,7,8] and diabetes [9] have been found to have worse outcomes.

Severe acute respiratory syndrome corona virus (SARS) and SARS-CoV-2 virus infect cells via angiotensin converting enzyme 2 (ACE-2) receptors [5,10,11]. ACE- 2 receptors are abundant within the pancreas and patients with SARS have been found to have elevated glucose levels compared to patients with pneumonia from other causes [11]. Patients with COVID -19 have been found to more commonly present with ketosis and diabetic ketoacidosis (DKA) [12], and there are case reports of COVID -19 patients presenting with new onset diabetes, DKA, and hyperglycemic hyperosmolar syndrome (HHS) [12,13].

Elmhurst Hospital Center, a 545-bed community hospital, part of the NYC Health and Hospital (NYC H&H) system and affiliated with the Icahn School of Medicine, serves a diverse community that is predominantly Hispanic, and with large populations of Asians and South Asians. SARS-COV-2 virus spread rapidly through this community, resulting in 1450 patients admitted to our institution with COVID-19 from March 1, 2020 to May 31, 2020, of whom 571 had diabetes.

We compared the clinical characteristics of patients with DKA with and without COVID-19 admitted during the three-month periods of March 1 to May 31 in 2019 and 2020 to see if there were any differences in these patient populations. For the COVID-19 patients, we evaluated whether outcomes correlated with the following: clinical characteristics, diabetes type, prescribed diabetic medications, use of beta blockers, angiotensin converting enzyme inhibitors (ACE), angiotensin receptor blockers (ARB), severity of COVID-19 at admission as reflected by inflammatory marker C-reactive protein (CRP), and the initial oxygen saturation.

## Materials and methods

Research design and participants

A retrospective study which included patients with diabetes admitted to Elmhurst Hospital Center in Queens, New York with a diagnosis of DKA with and without COVID-19 between March 1, 2020 and May 31, 2020 and a pre-COVID-19 group of DKA patients admitted from March 1 to May 31, 2019 was performed. Patients less than 18 years of age and pregnant women were excluded from the study. Institutional Review Board approval from Icahn School of Medicine was obtained.

Measurements and definition

COVID-19 diagnosis was based on clinical presentation and positive PCR testing. Chart review identified patients as having diabetes based on history and of new onset diabetes if they presented with DKA with no prior history of diabetes. Patients were included if they had DKA at the time of admission, defined by a pH <7.32, along with an anion gap >16, a concurrent glucose >200 mg/dl, and ketones present in blood or urine. Patients were also defined as having DKA if they met all the above criteria without measured ketones (ordered but not performed, neglected to be sent), but the primary team treated the patient for DKA and an endocrine reviewer agreed that the patient likely had DKA based on labs values and the clinical picture. DKA was further characterized by pH as mild (pH 7.25-7.32), moderate (pH 7.00-7.24) or severe (pH <7.00). For glucose >600 mg/dl (33.3 mmol/L) at admission, effective plasma osmolality was calculated, and if it was >320 mOsm/kg, the patient was considered to have HHS.

Data collection

Epic EMR software using ICD-10 codes was used to identify admitted patients in 2019 with a diagnosis of DKA; in 2020 three groups were identified: COVID-19 and diabetes (but no DKA), COVID-19 with DKA, and DKA without COVID-19. Patients from 2020 with DKA but not admitted for COVID-19 did not undergo routine PCR testing. All patients, even if PCR negative, had their charts reviewed by an infectious disease attending to assure that the clinical picture was not consistent with COVID-19 (no viral syndrome, lung infection, radiologic imaging consistent with COVID-19 infection, or gastroenteritis). All patients identified as having DKA had their charts reviewed to confirm that they met our DKA criteria. Mortality was defined if patient died during the admission.

Demographic variables included age, sex, ethnicity, and body mass index (BMI). Laboratory values used from first day of admission: glucose, anion gap, lactate, venous pH, ketones (blood or urine), CRP. Ketones were also used from second day of admission if not sent on the first day. Hemoglobin A1c (HbA1c) was included if it was sent anytime during the first week of the admission. The lowest oxygen saturation level noted during the first day of admission was used (found in either the emergency medical service record, emergency department vital signs, or from history in the medicine admission note). Medications collected as part of the study included insulin, metformin, secretagogues (sulfonylureas and glinides), dipeptidyl peptidase-4 inhibitors (DPP-4), glucagon-like-peptide-1 agonists (GLP-1), sodium-glucose- cotransporter-2 inhibitors (SGLT-2), betablockers, angiotensin converting enzyme inhibitors (ACE), and angiotensin receptor blockers (ARB).

Statistical analysis

Descriptive data are presented as frequencies with percentiles or ranges in patients with DKA stratified by COVID-19 status. Categorical variables were compared using Chi-square or contingency tables. Continuous variables were compared using ANOVA or non-parametric tests as appropriate. Our primary outcome was death. A two-sided p-value of less than 0.05 was considered significant. All statistical analyses were performed with the Statistical Package for Social Sciences, version 27 (SPSS, IBM, Chicago, IL).

## Results

Between March 1 and May 31 2020, 497 patients were admitted with COVID-19 and diabetes (without DKA), of which 194 (39%) died. In the same time frame, 35 patients were admitted with DKA and COVID-19, whereas 22 patients were admitted with DKA but without COVID-19 infection. During the same period in 2019, 25 patients were admitted with DKA. The percentage of patients with missing ketones was similar amongst the groups (p=0.627). Hispanic patients, males, and type 2 diabetes predominated in all the groups. Comparing COVID-19 with DKA, to the DKA patients without COVID-19 in both 2019 and 2020, COVID-19 patients were older (58.6 + 18.8 years v 48 +17.4 years, p=0.010), more likely to have hypertension (65.7% v 29.8%, p= 0.002), and had more severe lactic acidosis (p=0.006). There was a trend for COVID-19 patients to present with more severe DKA (p=0.062), and to be on an ARB (20% v 4.3%, p=0.050). There was no difference in use of beta blockers, ACE, or of diabetic medications. The percentages of patients admitted with glucose over 600 mg/dl (33.3 mmol/L) or HHS was similar (Tables 1, 2).

**Table 1:** DKA comparison: Groups 1 &2 are COVID-19 negative and group 3 is COVID-19 positive

Table 1. DKA Group Comparison Groups 1&2 COVID-19 negative and Group 3 COVID-19 positive								
		Age	BMI	HbA1c % (mmol/mol)	Glucose mg/dl, (mmol/L)	Anion Gap	pH (VBG)	Lactic Acid mmol/L
Group 1 2019	N	25	25	21	25	25	25	25
	Mean	47.1	26.5	11.8 (105)	679(37.7)	29.8	7.2	4.3
	Minimum	23.0	16.7	7.4 (57)	317(17.6)	16.0	7.0	1.0
	Maximum	89	37.9	17.7 (170)	1303 (72.4)	46.0	7.3	17.0
Group 2 2020	N	22	20	16	22	22	25.0	25.0
	Mean	49.1	25.9	12.8 (116)	587(32.6)	30.6	7.2	4.9
	Minimum	20.0	18.6	7.0 (53)	274 (15.2)	17.0	6.8	1.3
	Maximum	92	40.6	18.0 (173)	1500 (83.3)	50.0	7.3	14.4
Group 3 2020 (COVID-19)	N	35	34	26	35	35	35	35
	Mean	58.6	27.5	12.3 (111)	624 (34.7)	30.3	7.1	6.0
	Minimum	23.0	20.3	8.1 (65)	219 (12.2)	18.0	6.8	1.8
	Maximum	86	35.2	16.6 (158)	1471(81.7)	46.0	7.3	17.0
Three group comparison	p-value	0.033^†^	0.513*	0.617*	0.316^†^	0.806*	0.924^†^	0.015^†^
Groups 1 & 2 versus Group 3	p-value	0.009^‡^	0.274*	0.950*	0.786^‡^	0.796*	0.080^‡^	0.006^‡^
*ANOVA; ^†^Kruskal-Wallis Test; ^‡^ Mann-Whitney Test								

**Table 2:** DKA group comparison: Groups 1 &2 are COVID-19 negative and Group 3 is COVID-19 positive

Table 2. DKA Group Comparison Groups 1&2 COVID-19 negative and Group 3 COVID-19 positive						
		Group1 2019	Group 2 2020	Group 3 2020 COVID-19	Three group comparison	Groups 1&2 versus Group 3
Total Patients N (%)		25	22	35	p-value	p-value
DM Type	New onset	6(24.0)	4(18.2)	6(17.1)	0.251*	0.092*
	Type1	7(28.0)	5(22.7)	3(8.6)		
	Type2	12(48.0)	13(59.1)	26(74.3)		
Sex	Female	10(40.0)	8(36.4)	8(22.9)	0.32*	0.137*
	Male	15(60.0)	14(63.6)	27(77.1)		
Ethnicity	Hispanic	19(76.0)	11(50.0)	24(68.6)	0.097*	0.341*
	Asian &S. Asian	2(8.0)	2(9.1)	6 (17.7)		
	Other	2 (8.0)	1(4.5)	2(5.7)		
	Caucasian	2(8.0)	8 (36.4)	3(8.6)		
Hypertension	Yes	10 (40.0)	5(22.7)	23(65.7)	0.005*	0.002*
Glucose >600 mg/dl (>33.3mmol/l)		12(48.0)	7(33.3)	14(40.0)	0.597*	0.906*
HHS (Osm >320)		3 (12.0)	2(9.5)	6(17.1)	0.696*	0.414*
DKA Severity	Mild	12(48.0)	11(50.0)	12(34.3)	0.062^†^	0.044^†^
	Moderate	12(48.0)	9(40.9)	15(42.9)		
	Severe	1(4)	2(9.1)	8(22.9)		
*Pearson Chi-Square;^ †^Linear-by Linear Association						

Overall mortality for DKA patients with COVID-19 was 57% whereas DKA patients without COVID-19 had a mortality of 2.1% (p=0.0001); patients with diabetes and COVID-19 (but without DKA) had a mortality of 39% (p=0.035). Within the COVID-19 group, older age (mean age 65) (p=0.014), elevated CRP (p=0.012), low O_2_ saturation (p=0.001), and beta blocker use (p=0.01) were also associated with increased mortality. However, history of hypertension or the use of ACE or ARB were not associated with increased mortality. There was no association of BMI, severity of DKA, anion gap, lactic acidosis, presence of HHS, or particular diabetes medications with mortality (Tables 3, 4).

**Table 3:** COVID-19 associations with Mortality

Table 3. COVID-19 Associations with Mortality					
		N	Mean	Std. Deviation	p-value
Age	Alive	15	50.3	15.6	0.022*
	Deceased	20	64.8	19	
BMI	Alive	14	27.6	4.3	0.883*
	Deceased	20	27.4	3.6	
HbA1c % (mmol/mol)	Alive	14	12.9 (117.5)	2.5 (3.8)	0.099*
	Deceased	14	11.5 (102.2)	2.5 (3.8)	
Glucose mg/dl (mmol/L)	Alive	15	645.3 (35.8)	331.7 (18.4)	0.934^†^
	Deceased	20	607.8 (33.7)	224.1 (12.4)	
Anion Gap	Alive	15	31.9	6	0.292*
	Deceased	20	29.2	8.1	
pH (VBG)	Alive	15	7.12	0.18	0.908^†^
	Deceased	20	7.15	0.15	
Lactic Acid mmol/L	Alive	15	5.96	2.9	0.610^†^
	Deceased	20	6.07	4.16	
CRP mg/L	Alive	15	124.6	108.6	0.012^†^
	Deceased	19	218.8	81.8	
O_2_ Saturation	Alive	15	90%	16%	0.001^†^
	Deceased	19	73%	20%	
*ANOVA; ^†^Mann-Whitney U test.					

**Table 4:** COVID-19 patients associations with Mortality

Table 4. COVID-19 Patients Associations with Mortality				
Total Patients N (%)		Alive: 15	Deceased:20	p- value
DM Type	New onset	3 (20.0)	3 (15.0)	0.60*
	Type1	2 (13.3)	1(5.0)	
	Type2	10 (66.7)	16 (80.0)	
Sex	Female	3 (20.0)	5 (25.0)	1.00^†^
	Male	12 (80.0)	15 (75.0)	
Hypertension	No	7 (46.7)	5 (25.0)	0.28^†^
	Yes	8 (53.3)	15 (75.0)	
Glucose > 600 mg/dl (>33.3 mmol/L)		6 (40.0)	8 (40.0)	1.00^†^
Osm > 320 mOsm/kg		2(13.3)	4 (20.0)	0.68^†^
DKA Severity	Mild	5 (33.3)	7 (35.0)	0.40*
	Moderate	5 (33.3)	10 (50)	
	Severe	5 (33.3)	3(15.0)	
Medication History		Alive :13	Deceased:17	
Insulin		7 (53.8)	9 (52.9)	1.00^†^
Metformin		4 (30.8)	6 (35.3)	1.00^†^
SGLT-2		1 (7.7)	0	0.43^†^
GLP-1		0	0	
DPP-4		0	3 (17.6)	0.24^†^
Insulin Secretagogues		1 (7.7)	4 (23.5)	0.36^†^
ACE		5 (38.5)	3 (17.6)	0.24^†^
ARB		2 (15.4)	4 (23.5)	0.67^†^
Beta Blockers		0	7 (41.2)	0.010^†^
*Pearson’s Chi-Square; ^†^Fisher’s Exact test				

## Discussion

We found over a doubling of admissions for DKA during the 2020 review period largely due to increase in patients with type 2 diabetes presenting with COVID-19, and patients presenting with both DKA and COVID-19 exhibited a significantly higher mortality than patients with either disease alone. The COVID-19 patients were significantly older, had more hypertension, and had more severe DKA (pH<7.0) and lactic acidosis on presentation than DKA patients without COVID-19. There was a male predominance which was similar to our patients without COVID-19 and there was no difference in BMI. We did not find higher glucose levels or more HHS within the COVID-19 group.

There are a number of possibilities for the increase in rate of DKA seen among patients in the COVID-19 group. High cytokine levels seen in COVID-19 could lead to beta cell dysfunction and possible apoptosis as well as an increase in insulin resistance in both liver and muscle cells [14]. SARS-CoV-2 has been shown to bind ACE receptors in endocrine pancreas and the damage to islets could contribute to hyperglycemia [10,15]. However, a recent study of the SARS-COV-2 virus showed binding of the virus to exocrine pancreas but not to the islet cells indicating that the virus may not cause damage to the beta cells directly. SARS-CoV-2 has also been shown to infect cells of the small intestine which could lead to disruption of incretins, glucagon like peptide -1 (GLP-1) and gastrointestinal peptide (GIP), which could further adversely affect the ratio of insulin to glucagon [16]. A combination of all these factors may lead towards a more ketotic state as well as DKA in those who are genetically predisposed.

Within the COVID-19 DKA group, the death rate was extremely high compared to patients with diabetes and COVID-19, or DKA patients without COVID-19. Our study confirms the higher death rate found in COVID-19 patients with DKA as well as the association with older age as reported by Kishore et al. [17]. We did not find an association with higher BMI. Alkundi et al. found that COVID-19 patients presenting with DKA had better outcomes as compared to patients without DKA [1]. This discrepancy could possibly be due to difference in sample size, ethnicity, and severity of the COVID-19. For example, our patient population may have more ketosis-prone patients with type 2 diabetes, patients with hypertension, and patients with more severe lactic acidosis.

There were no indicators of diabetes other than DKA itself which were associated with mortality. Not surprisingly, low O_2_ saturation and high CRP, indicators of severe COVID-19 infection, were associated with mortality. Many of the medications prescribed for diabetes increase ACE-2 levels; it is not clear if their use accounts for the worse outcomes of patients with diabetes [15]. There are also reports of renin-angiotensin inhibitors improving outcomes in COVID-19 patients with hypertension [18]. In our patients, there was no correlation with use of ARB and ACE with mortality. Surprisingly however, prior use of beta blockers was associated with an increase in mortality. It is possible that beta blocker use may reflect underlying cardiovascular disease.

The limitations of our study were small sample size, limited medical histories due to severity of illness causing early intubation, and lack of standardization of treatment for COVID-19, cytokine storm, acute respiratory distress syndrome, and thrombosis.

## Conclusions

Based on our study, the demographics of the COVID-19 patients are similar to our patients without COVID-19 - predominantly male, Hispanic, and with type 2 diabetes. The COVID-19 patients were older, had a history of hypertension, and have more severe DKA and lactic acidosis on presentation than patients without COVID-19. There was no increase in DKA with HHS. DKA by itself predicted a poor outcome for COVID-19 patients, with a 57% mortality rate; however, there was no association with the parameters for diabetes, BMI, or hypertension. Rather, mortality was primarily driven by the severity of COVID-19 infections at presentation as indicated by high CRP levels and low O_2_ saturation. We did not find an association with hypertension, but did find a surprising association between beta blocker use and mortality. Further research is warranted to understand the mechanisms of how SARS-CoV-2 infection leads to increase in ketosis and diabetic ketoacidosis. Our findings will need to be confirmed in a larger database.
